# BCRgt: a Bayesian cluster regression-based genotyping algorithm for the samples with copy number alterations

**DOI:** 10.1186/1471-2105-15-74

**Published:** 2014-03-15

**Authors:** Shengping Yang, Xiangqin Cui, Zhide Fang

**Affiliations:** 1 Biostatistics Program, School of Public Health, LSU Health Sciences Center, 2020 Gravier Street, New Orleans, LA 70115, USA; 2 Department of Pathology, School of Medicine, Texas Tech University Health Science Center, Lubbock, Texas, USA; 3 Department of Biostatistics, University of Alabama at Birmingham, 1665 University Blvd, Birmingham, AL 35294, USA

**Keywords:** Bayesian cluster regression, Copy number alteration, Genotyping, SNP array

## Abstract

**Background:**

Accurate genotype calling is a pre-requisite of a successful Genome-Wide Association Study (GWAS). Although most genotyping algorithms can achieve an accuracy rate greater than 99% for genotyping DNA samples without copy number alterations (CNAs), almost all of these algorithms are not designed for genotyping tumor samples that are known to have large regions of CNAs.

**Results:**

This study aims to develop a statistical method that can accurately genotype tumor samples with CNAs. The proposed method adds a Bayesian layer to a cluster regression model and is termed a Bayesian Cluster Regression-based genotyping algorithm (BCRgt). We demonstrate that high concordance rates with HapMap calls can be achieved without using reference/training samples, when CNAs do not exist. By adding a training step, we have obtained higher genotyping concordance rates, without requiring large sample sizes. When CNAs exist in the samples, accuracy can be dramatically improved in regions with DNA copy loss and slightly improved in regions with copy number gain, comparing with the Bayesian Robust Linear Model with Mahalanobis distance classifier (BRLMM).

**Conclusions:**

In conclusion, we have demonstrated that BCRgt can provide accurate genotyping calls for tumor samples with CNAs.

## Background

Most multicellular organisms have two copies of chromosomes (diploid organisms). An allele refers to one of the different arrangements of nucleus acids in the same genetic location (locus) on homologous chromosomes. Every individual has two alleles in one locus, making up the genotype, either homozygous or heterozygous. A Single Nucleotide Polymorphism (SNP) is a genetic variation where one single nucleotide differs between the two alleles in members of a population or paired chromosomes. In humans, although most SNPs have no effect on health, some of them have been shown to affect how an individual develops diseases and responds to drug treatments. As a result, a great deal of contemporary biomedical research focuses on investigating the association between SNPs and phenotypes (e.g. diseases), known as Genome-Wide Association Study (GWAS).

Since the release of the first commercial SNP array in 1996 by Affymetrix (Santa Clara, CA, USA), SNP arrays have been intensively used in GWAS. In the last decade, these applications have resulted in the identification of a large number of common genetic variants that are associated with different diseases. Today, while Next-Generation Sequencing (NGS) has greatly changed the landscape of genetic research due to the rapid drop in cost, SNP array technique is still more economical to use and has many other advantages, such as less labor intensive sample preparation and being more conducive to working for a large number of samples.

Accurate SNP genotyping is one of the important factors of a successful GWAS. This has motivated researchers, in both academia and industry, to develop sophisticated algorithms to improve genotyping accuracy.

For technical supports, Affymetrix developed the accompanying genotyping software - Modified Partitioning Around Medoids (MPAM), after the release of their first generation GeneChip array, and then the Dynamic Model (DM) algorithm for the GeneChip 100 K array. These two arrays have similar designs although the 100 K arrays include more SNPs. For each array, every SNP is interrogated by a number of probe quartets, each of which is composed of a 25-base-pair Perfect Match oligonucleotide probe (PM; probe sequence matching the target sequence) and a Mismatch probe (MM; probe sequence obtained by replacing the middle (13th) base of the PM with its Watson-Crick complement) for alleles A and B separately. To make a genotype call for a SNP, one can use allelic intensities detected by the PM probe(s) as foreground and those by the MM probe(s) as background to calculate the likelihood for each possible genotype (AA, AB, BB, or NoCall), and then make the genotyping call, based on the highest likelihood [1]. For example, if the likelihood for genotype AA is the largest, we assign “AA” as the genotype for the SNP. However, since both MAPM and DM were designed to genotype one SNP on one chip (sample) at a time, their overall genotyping call accuracies are not satisfactory. To improve genotyping accuracy, many other methods have been proposed, including the Robust Linear Model with Mahalanobis distance classifier (RLMM) [2]. Specifically, for each SNP, by using PM probes only (it has been shown that MM probes can cause more trouble than good), RLMM includes all allelic intensities across multiple chips in a model to estimate the possible genotype clusters. In this way, the between-chip-level variations can be well accommodated. Subsequently, an improved model, named BRLMM [3], was proposed by adding a Bayesian step to RLMM. BRLMM utilizes the genotyping calls from DM as the initial “seed” genotypes to determine the parameters of the Bayesian prior distributions, and adopts the multi-array model approach to enhance the accuracy of genotype cluster estimates. Note that the link between BRLMM and DM limits the application of BRLMM to newer generations of arrays, such as SNP array 5.0/6.0, because these new arrays do not have MM probes that are employed by DM to make the initial “seed” genotype calls. To overcome this limitation, BRLMM-P was introduced, which estimates initial “seed” genotypes directly from the clustering properties of the PM probes [4]. However, this data-driven approach requires a rigorous performance standard for SNP screening. As a result, for 500 k array, only 440,794, out of 500,568, SNPs are considered to have high quality BRLMM-P calls.

The Birdsuite software, introduced by the Broad Institute in collaboration with Affymetrix, was initially developed for genotyping SNP 6.0 array data. The birdseed, one component of the software, like all other software mentioned above, was developed exclusively for genotyping diploid genomic regions that do not have CNAs (duplication and deletion events occurring in somatic cells) [5], and copy number variations (CNVs; local duplication and deletion events occurring at kb or Mb scale in germline cells). The Birdseye software, another component of Birdsuite, although being able to detect rare CNVs and genotype SNPs in CNV regions, was not designed to make genotype calls for samples with CNAs. This is generally because of issues such as random occurrence of CNAs along the genome and contamination of normal tissues in tumor samples [6].

Like Affymetrix, Illumina has its own genotyping software, called GenCall and implemented within the BeadStudio, to genotype SNPs on the BeadChip arrays. Similar to RLMM and BRLMM-P, GenCall applies a multi-array approach and can make genotype calls with or without using a reference dataset, depending on the sample size.

In addition, other methods are also available for genotyping Affymetrix/Illumina arrays. These algorithms differ in their aims. For example, Corrected Robust Model with Maximum Likelihood Distance (CRLMM) [7] focused on reducing across-lab variations through improving data pre-processing. SNiPer-High Density (SNiPer-HD) [8] was designed to obtain high accuracy for highly informative (that is, the genotype is called with high confidence) SNPs. Chiamo [9] and ALCHEMY [10] were especially useful for calling small batch sizes and highly homozygous populations. Algorithms designed for Illumina arrays include GenoSNP [11] for improved calling on rare variants; M^3^[12], which is based on Modified Mixed Model; and Opticall [13] for more accurately genotyping rare, low-frequency and common variants.

Although the majority of these algorithms showed some success in improving the genotyping accuracy, they were initially developed for genotyping samples without CNAs. We comment that GenoCN [14] could genotype samples with CNAs, but it adopted a one chip at a time approach, and was designed mainly for Illumina array data.

On the other hand, many genetic diseases (cancers) are associated with CNAs, and lots of methodologies have been developed to correctly detect CNAs [15-17]. By each of these methods, copy number status (gain/loss/unchanged) can be obtained for every SNP in a sample. Generally, CNA calls for tumor samples with paired normal samples can have a high level of accuracy. If there are no paired normal samples, one can still be able to obtain reliable CNA calls by using a generic normal as control [18]. Note that since CNAs are somatic alterations, it is difficult to predict where on the chromosome CNAs occur. Meanwhile, in the sample preparing step, it is usually hard to avoid normal tissue contamination. Most cluster-based genotyping algorithms could run into the problem of misclassification at the regions of CNAs because CNAs substantially altered the number of A and B alleles, and thus, the A and B allele log-intensities from the array. We recommend incorporating CNA status into the cluster regression model to adjust for the effect of CNAs. To do so, both high quality copy number calls and stringent sample screening are necessary.

In this paper, we propose a Bayesian Cluster Regression based genotyping (BCRgt) approach to genotype samples with CNAs. This is motivated by the facts that cancer tissues often have large regions of genetic structural alterations such as CNAs [19], and that researchers have been interested in genotyping SNPs from such samples.

The setup of the paper is as follows. In Section Methods, we will describe the steps of BCRgt. In Section Results and discussion, we use HapMap (details in Subsection Genotyping the samples without CNAs by BCRgt) genotype calls as a gold standard to calculate the concordance rates, the probability that a pair of genotyping results have a certain genotype, given that one of the pair has such a genotype, in order to evaluate the performance of BCRgt for samples without CNAs. Furthermore, we will apply BCRgt to samples with CNAs, to illustrate the improvement that BCRgt makes in genotyping CNA regions comparing over BRLMM. At the end of the paper, we will briefly discuss the ideal situation for using BCRgt and the limitations of this method.

## Methods

There are several widely used Affymetrix SNP array platforms, including the genome-wide human SNP Array 5.0/6.0, GeneChip® Mapping 10 K/100 K/500 K Array. In this paper, we will focus on the Affymetrix GeneChip® Mapping 500 K array. The Array Set consis ts of Nsp and Sty arrays for Nsp I and Sty I restriction enzyme digested genomes, respectively. Each Nsp (or Sty) array has the capacity to interrogate about 250,000 SNPs, with, on average, 6 to 10 pairs of allele-specific intensity measurements per SNP. In our analysis, for each SNP, these 6 to 10 measurements are averaged for both A and B alleles, and the averaged values will be used as the input in BCRgt. Note that although Mapping 500 K array has both PM and MM probes for each allele, BCRgt utilizes only PM probe intensities, and thus, can be directly applied to newer generations of Affymetrix arrays.

### Quantile normalization

SNP-specific signal intensity distributions are different not only across SNPs but also across samples/labs/studies. SNP array genotyping is usually carried out array-wisely, one SNP at a time. Thus, a successful genotyping algorithm can benefit from an appropriate normalization step to adjust for the undesirable between-array experimental/biological variations. Quantile Normalization (QN) is one of the widely used normalization methods. By assuming that the distributions of DNA abundances are nearly the same across all samples, QN transforms the raw intensities to the corresponding quantile’s value. Although QN has many good properties when all the samples for genotyping do not have CNAs, it does not perform well in normalizing samples with CNAs [20]. Therefore, for simplicity we recommend applying QN on samples without CNAs, but not doing so for samples with CNAs.

### Bayesian cluster regression

Let *x*_*ij*_ ∈ (0, *∞*), *y*_*ij*_ ∈ (0, *∞*) denote the log-intensities of *A*, *B* alleles of the *j*^*th*^ SNP of the *i*^*th*^ subject. A successful genotyping approach should depend on the relative relationship between A and B alleles, not on the absolute values of A and B alleles. Thus, we will make genotype calls based on such allelic relationship, and arbitrarily choose either A or B allelic log-intensity as the predictor so that the linear relationship between A and B alleles can be investigated via a traditional linear regression if they come from the same population (genotype). We further assume, for simplification, that the allelic log-intensities of adjacent SNPs are independent of each other. The rationale underlying this assumption is that the physical distance between two consecutive SNPs is very often at least hundreds of base pairs apart. For notational convenience, we will drop the subscript *j* because genotyping call is made independently for each SNP in the proposed algorithm. In addition, for every SNP, both A and B allelic log-intensities are centralized at the median of the A allele log-intensities.

#### Traditional cluster regression

Consider the case of three clusters, and assume that the paired observations (*x*_*i*_, *y*_*i*_) come from one of the three unknown component populations (clusters), that is, *C*_*i*_ = *k* with probability *π*_*k*_, where *k* ∈ {1, 2, 3}. Let ***X*** be a matrix with the first column being an all-ones vector, and the second column consisting of the corresponding *x* values from the cluster *k*. For a given cluster *C*_*i*_ = *k*, the relationship between ***X*** and *y* can be expressed in the following model with parameter **β**_k_,

$$y_{C_{i}=k}=X_{C_{i}=k}\beta_{k}+\xi_{k},$$

where *C*_*i*_ ~ *Multinomial*(*π*)  and the error vector *ξ*_*k*_ ~ *N*(0, *σ*^2^*I*) [21]. Note that the three clusters represent three different genotypes, AA, AB, BB, and that each of the three linear regression models in the above equation is fitted for one of the three genotypes. A NoCall can be made if none of the probability of AA, AB or BB is above a certain threshold, such as 99.9%. We comment that setting such a threshold can improve the overall genotyping accuracy, but, as a tradeoff, the overall call rate (the percentage of SNPs that are called as AA, AB or BB rather than NoCall) drops.

#### Bayesian cluster regression

Despite the fact that cluster regression has many good properties in classifying samples into different groups at the individual SNP level, further improvement can be made by incorporating additional information from all SNPs across the samples. Adding a Bayesian step would be a good choice for this purpose.

For every SNP, cluster regression partitions all samples into “AA”, “BB” and “AB” genotype clusters. However, problems might arise if one or even two genotypes do not exist for all samples, thus misclassification is very likely to occur. BCRgt adds a Bayesian layer to pre-define the distribution of each of the three clusters based on the intercept and slope of each cluster, which are estimated from the A and B allele log-intensities of a large sample of all SNPs and all samples. Then, if the genotypes of a SNP are the same for all the samples, applying a prior distribution based on these estimates will improve the accuracy in estimating the parameters of the posterior distributions.

One of the key issues in a Bayesian model is to derive the posterior distribution. In order to have a closed-form posterior distribution, the commonly used prior for (**β**, σ^2^) is a normal-inverse-gamma distribution of the form *p*(**β**, *σ*^2^) = *p*(**β** | *σ*^2^)*p*( *σ*^2^) with the first part being a multivariate normal distribution and the second part an inverse-gamma distribution [21]. Note that the posterior distribution is also a normal-inverse-gamma distribution. In this paper, for model and computational simplicity, we will use the frequentist method (that is, parameters are estimated from the current data itself, no prior information is needed) to estimate *σ*^*2*^, instead of imposing a prior on *σ*^*2*^. By doing so, we impose that the prior is reduced to a multivariate normal distribution *N*(**β**_*prior*_, *σ*^2^**V**), where the elements *β*_*k*0,*prior*_ and *β*_*k*1,*prior*_ of the two-dimensional mean vector **β**_*prior*_ represent the expected values of the distributions of the intercept and slope for the cluster *k*, respectively, and *σ*^2^**V** is a diagonal matrix with the diagonal elements being the corresponding variances. The expectation of the posterior distribution can be easily derived as ${\hat{\beta }}_{\mathrm{Bayes}}={\left(V^{-1}+X'X\right)}^{-1}\left(V^{-1}\beta_{\mathrm{prior}}+X'y\right)$ (see Additional file 1). We comment that higher-order polynomial terms may be added to the model in order to better accommodate the samples with less stringent quality control. However, since minor changes in higher-order terms usually have substantial effects on model fitting, in order to make sure that the prior distributions of the higher order terms do not have dominating effects on the posterior distribution, a very strong prior favoring the null hypothesis, i.e., higher order terms do not contribute to the model, should be used. In practice, we observed that, for the samples we have tested, adding a quadratic term to the simple linear model could only result in a negligible difference in the fitting results. Thus, for the model parsimony reason, we only considered a simple linear model.

The Expectation-Maximization (EM) algorithm [22,23] was used to estimate **β**. Specifically, the expectation step was to find the expectation of *Q*(**β**, *σ*^2^|**β**^(*t*)^, *σ*^2(*t*)^), namely *E*(*C*_*ik*_ | *y*, **β**_*k*_, *σ*^2^), based on data; and the maximization step was to update (**β**, σ^2^) with (**β**^(t+1)^, σ^2(t+1)^) that maximizes *Q*(**β**, *σ*^2^|**β**^(*t*)^, *σ*^2(*t*)^) (see Additional file 1). The EM algorithm was converged/stopped when the difference of two consecutive estimates of the parameters was smaller than a pre-set small value or after 30 iterations. The parameter values from the last iteration were taken as the final estimates.

### The values of the parameters of the prior distributions

Setting parameter values for the prior distributions is a critical step in a successful Bayesian cluster regression. We describe in the next subsections how to setup these values.

#### The expectations of the intercepts and slopes of the prior distributions

**Figure 1:**
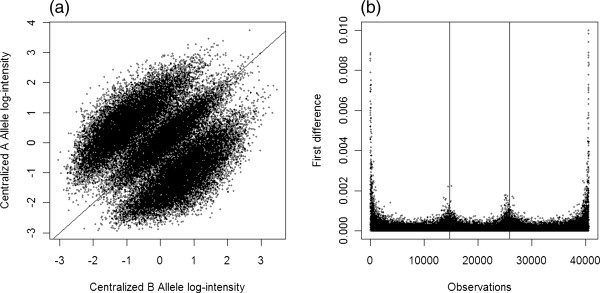
**Plots illustrate how parameters of the prior distributions are estimated. (a)** Scatter plot of Centralized A and B allelic log-intensities for 5,000 SNPs. Note that observations are symmetric about the 45 degree line. **(b)** First difference plot: the two vertical lines partition observations into three clusters corresponding to observations with AA, AB and BB genotypes.

We followed the approach in [13] to take advantage of the information obtained from the data. Specifically, we randomly selected a subset of all the paired A and B allelic log-intensities across samples. The size of the subset is quite arbitrary, for example, 5,000 to 50,000. The three clusters representing AA, AB and BB genotypes are clearly separated (Figure 1(a)) and can be roughly classified by most of cluster analysis methods proposed in literature. Our approach is a computationally simple alternative. We calculated the first difference statistics [24], *diff*(*x* - *y*) = (*x* - *y*)_(*t*)_ - (*x* - *y*)_(*t* - 1)_, where (*x-y*) is log-Ratio of the A and B allelic signal intensities and *t* is the order, and searched by a moving average (the un-weighted mean of the previous *n* data points) approach for the two maximum values, between the 25th and 50th percentiles and between the 50th and 75th percentiles, respectively. The numbers of observations separated by the two maximum values are roughly equal to the numbers of observations with AA, AB and BB genotypes respectively (Figure 1(b)). After identifying these three clusters, we fitted a simple linear regression for each cluster and used the estimated model parameters as the intercept and slope of the prior distribution for the corresponding cluster. We comment that, 1) in practice, although the estimates of the slopes are somewhat different from 1 (45 degree), it usually works well to use 1 for all slopes for simplification purpose; 2) the proportion of observations with AB genotype is lower than those of observations with AA/BB genotypes, and this can be conveniently adjusted in the EM algorithm by putting different weights for AB and AA/BB genotypes.

#### The variances of the intercepts and slopes of the prior distributions

It is well known that the posterior mean of the parameter vector in Bayesian linear regression can be expressed in terms of the least square estimate and the prior mean. To obtain the variance of the least square estimate, we chose 100 SNPs that have only one genotype (either AA or BB), and calculated the average of the 100 variances of the least square estimates of the slope. We then used one third of such an average value as the variance of the prior distribution for the slope to ensure that the prior information on the slope plays a substantial role. As a justification, we replaced one third by a decreasing sequence: one fifth, one tenth of the average value, and observed very small differences in genotype calling results (data not shown). Similarly, we set the variance of the prior distribution for the intercept to be roughly equal to three times of that estimated from the data so that the intercepts are more influenced by the data.

Meanwhile, the model in maximization step is defined as

$$\begin{array}{l} y_{C_{i}=k}=\beta_{k0}+x_{C_{i}=k}\beta_{k1}+\xi_{k},\\ \mathrm{where}\;k=1,2,3,\mathrm{and}\;\xi_{1}=\xi_{3}=\sqrt{2\xi_{2}}. \end{array}$$

The error term for the heterozygous AB cluster was set smaller than those of the homozygous clusters in order to adjust for the difference in the spreads of the heterozygous and homozygous observations (Figure 1). The adjustment factor could be obtained empirically from the sample data described in Section Genotyping the samples without CNAs by BCRgt. Here we use  for convenience.

**Figure 2:**
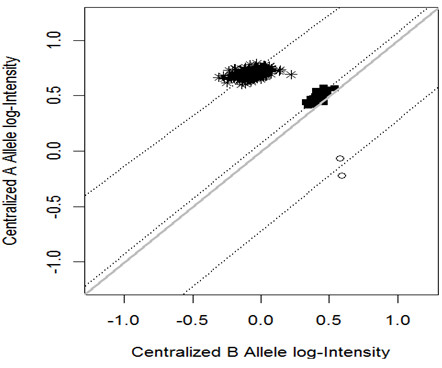
**An example of applying BCRgt to genotype calling on one SNP.** Three clusters represent three genotypes (AA, AB and BB are represented by star “*”, solid square and circle, respectively). The bold black line is the 45 degree line, and the three dotted lines are the prediction lines for three clusters separately.

For demonstration purposes, we applied BCRgt to a good-quality SNP with no missing genotypes and presented the results in Figure 2. The effect of the priors can be seen from two observations with BB genotype (circles). The prediction line would go through both dots if no prior were imposed. With BCRgt, the slope for BB genotype is very close to 1 (45 degree line). This example shows how the prior information on slope works. And we expect that the true slope for BB genotype would be approximately 1 if more samples were observed. In addition, the heterozygous cluster (solid squares) has less spread than the cluster representing AA genotype (symbol “*”), which concurs with the pattern in Figure 1. We also demonstrated in Additional file 1: Figure S1 that BCRgt performed well if one or two genotypes were missing.

### Incorporating the CNA variable into the model

Comparing with the commonly used genotyping methods (cluster-based models), BCRgt has the advantage of conveniently adding extra covariates in the model. Next, we will explain how to utilize this advantage in incorporating copy number information in BCRgt. The reason for adding this information is given in Section Background.

Once the CNA calls are obtained, we include CNA as a covariate in the regression, and the maximization step will be to find the maximum likelihood estimators for the following new regression model. The main rationales behind this model are that the relationship between the log-intensities of A, B alleles within a cluster (genotype) is approximately linear (see the left panel of Figure 1), and that any allele gain/loss will move the corresponding observation vertically.

$$\begin{array}{l} y_{C_{i}=k}=\beta_{k0}+x_{C_{i}=k}\beta_{k1}+CN_{C_{i}=k}\beta_{k_{\mathrm{CN}}}+\xi_{k},\\ \mathrm{where}\;k=1,2,3,\mathrm{and}\;\mathrm{CN}\;\mathrm{is}\;\mathrm{defined}\;\mathrm{as}\\ \begin{array}{ll} \mathrm{CN}=\;0, & \mathrm{if}\;\mathrm{no}\;\mathrm{copy}\;\mathrm{number}\;\mathrm{change},\\ \mathrm{CN}=‒\mathrm{constant}, & \mathrm{if}\;A\;\mathrm{allele}\;\mathrm{loss},\\ \mathrm{CN}=\;\mathrm{constant}, & \mathrm{if}\;B\;\mathrm{allele}\;\mathrm{loss},\\ \mathrm{CN}=‒\mathrm{constant}/5, & \mathrm{if}\;B\;\mathrm{allele}\;\mathrm{gain},\\ \mathrm{CN}=\;\mathrm{constant}/5, & \mathrm{if}\;A\;\mathrm{allele}\;\mathrm{gain},\\ \mathrm{with}x_{C_{i}=k}\mathrm{and}y_{C_{i}=k} & \mathrm{being}\mathrm{the}\log‒\mathrm{intensities}\;\mathrm{of}\;A\\ & \mathrm{and}\;B\;\mathrm{alleles},\mathrm{respectively}. \end{array} \end{array}$$

Note that, for samples with copy number loss, if the log-intensity of A allele is greater than that of B allele, we define them as B allele loss; otherwise, we define them as A allele loss. A or B allele gain can be defined similarly for the samples with copy number gain. In EM algorithm, we treated *CN* as a fixed factor for computational simplification. Also, for copy number gain based on empirical evaluation, the input value for *CN* is five times smaller than that for copy number loss in order to adjust for 1) the saturation effect – the maximal signal intensity is limited by the amount of corresponding probes on the array, so that increases in copy number might not be proportionally reflected in signal intensity; and 2) the difference in absolute change in signal intensity between copy number gain and loss (see Additional file 1: Figure S2). In addition, we used the larger of the absolute values of the 2.5 and 97.5 percentiles of the differences of A and B allele log-intensities as the input value for “constant” in the above model. We comment that, because we choose a weak prior on $\beta_{k_{\mathrm{CN}}},$ these pre-set values do not significantly affect genotyping results unless they are completely out of range.

## Results and discussion

### Genotyping the samples without CNAs by BCRgt

In order to evaluate the performance of our proposed method, a decent number of samples with known genotypes are needed. The International HapMap Consortium has made such samples available – the Phase 2 of HapMap data release provides reference calls for approximately 70% of the SNPs on the GeneChip® Human Mapping 500 K Array Set for a collection of 270 samples, and the Phase 3 release provides more reference calls. These calls were generated from the consent of results from various technologies and sources, and are generally considered as a gold standard, although it is reasonable to assume that the genotype calls in HapMap still have some rare errors.

The Mapping 500 K Array data for all 270 HapMap samples were downloaded from the NCBI’s Gene Expression Omnibus website (Accession number: GSE5173). In the comparison, we focused on chromosome 1 to demonstrate the performance of BCRgt, but it is reasonable to assume that results from other autosomal chromosomes would be very similar.

Genotype calls for over 93% of SNPs on chromosome 1 are available from the HapMap genotype data (July 2009 release, http://www.HapMap.org). Concordance rates, the percentage of agreement between HapMap calls and BCRgt calls, for different datasets are presented in Table 1. There are two sections in this table. The upper section (datasets from A to G) displays genotyping concordance rates using the BCRgt without any model training, that is, we used generic prior distributions for every SNP. Note that in this upper section we genotyped different numbers of samples (270 samples for A; 50 for B, C, D; and 30 for E, F, G) in order to evaluate the effect of sample size on concordance rate. Also, for datasets A to D, H, each dataset has two rows corresponding to two different call rates. The lower section (datasets from H to O) displays the concordance rates using BCRgt with a model training step. We randomly split the whole HapMap 270 samples into two groups of equal size (135 each), and used one group as the training set to obtain parameter estimates (including intercepts and slopes) of the prior distributions for each SNP. Then we employed these prior distributions in BCRgt, and performed genotyping on different subsets of samples (with size 135, 50, 30 and 20) randomly selected from the other group. We expected that, by including the training step, much higher concordance rates with HapMap genotype calls can be achieved.

**Table 1:** Call rates and concordance rates for various datasets of different sizes (values are in percentages)

**DataSet**	**# of Samples**	**Overall call rate**	**Homo call rate**	**Hetero call rate**	**Overall concord rate**	**Homo concord rate**	**Hetero concord rate**
A	270	**99.56**	99.80	98.97	**99.59**	99.58	99.63
A	270	**100.0**	100.0	100.0	**99.50**	99.51	99.46
B	50	**99.56**	99.82	98.82	**99.58**	99.58	99.57
B	50	**100.0**	100.0	100.0	**99.47**	99.52	99.35
C	50	**99.73**	99.90	99.49	**99.58**	99.61	99.27
C	50	**100.0**	100.0	100.0	**99.52**	99.58	99.35
D	50	**99.53**	99.79	98.89	**99.44**	99.41	99.51
D	50	**100.0**	100.0	100.0	**99.33**	99.34	99.31
E	30	**99.61**	99.84	98.92	**99.35**	99.43	99.13
F	30	**99.71**	99.89	99.24	**99.43**	99.21	99.51
G	30	**99.56**	99.79	98.92	**99.25**	99.29	99.13
H	135	**99.51**	99.76	98.94	**99.77**	99.83	99.61
H	135	**100.0**	100.0	100.0	**99.70**	99.77	99.52
I	50	**100.0**	100.0	100.0	**99.65**	99.71	99.51
J	50	**100.0**	100.0	100.0	**99.61**	99.68	99.43
K	50	**100.0**	100.0	100.0	**99.76**	99.82	99.61
L	30	**100.0**	100.0	100.0	**99.65**	99.71	99.50
M	30	**100.0**	100.0	100.0	**99.64**	99.66	99.60
N	30	**100.0**	100.0	100.0	**99.56**	99.61	99.41
O	20	**100.0**	100.0	100.0	**99.49**	99.50	99.44

For dataset A, the overall concordance rates between HapMap and BCRgt genotypes are 99.59% and 99.50% with overall calling rates at 99.56% and 100%, respectively. We can see that the lower call rate is associated with a higher concordance rate because SNPs that are difficult to call were treated as NoCall, and this reduced the probability of making an error. For datasets B, C and D, each having 50 samples randomly selected from the HapMap samples, the average overall concordance rates are 99.53% and 99.44% with average calling rates at 99.60% and 100%, respectively. With further reduced size of 30, datasets E, F and G have the average overall concordance rate of above 99.34% with an average overall call rate of 99.63%. We observed that the concordance rate declines as the dataset becomes smaller. Note that this is an empirical conclusion from several estimations. It would be interesting to rigorously assess the statistical relationship between sample size and concordance rate in the future study.

The percentages in the lower section of Table 1 indicate that adding the training step can greatly improve genotyping accuracy. For example, the overall concordance rates (dataset H) reach 99.70% and 99.77% with overall call rates at 100% and 99.51%, respectively. For a further reduced sample size of 20, we could still have an overall concordance rate at about 99.5% (dataset O). In Table 1, we also list concordance rates for homozygous and heterozygous calls separately, which are generally close to the overall concordance rates.

We could not provide the concordance rate for dataset of size greater than 270 due to the sample size limitation in the HapMap database. Interested users are encouraged to apply the strategy described in [25] for genotyping larger datasets.

Our training step is different from the commonly used approach in that the latter would use the whole set of the 270 samples from one laboratory to train the model, and then perform genotyping on another independent dataset from a different laboratory (the same 270 samples). We comment that, although not taking advantage of the whole HapMap samples in the training step, our method can avoid the possibility of inflating the measures of performance because it does not use the same biological samples in both the training and validating sets. Note that if the objective is to genotype samples other than those participated in the HapMap study, we will use all the 270 samples as the training set.

### Genotyping samples with CNAs by BCRgt

Genotyping samples with CNAs is more challenging, and the validation of genotypes for such samples is currently difficult due to the lack of a gold standard such as the HapMap genotype data. Therefore, in this subsection, we will provide some individual SNP level as well as individual sample level examples to illustrate how BCRgt performs. The comparisons, regarding heterozygous call rate and genotyping error rate, between genotype calls made by BCRgt and BRLMM, will be presented at the end of this subsection.

The data, downloaded from GEO (access#: GSE21349), were generated for the study of myeloma, a disease associated with CNAs [26]. There are 80 paired myeloma samples in the dataset. The rationales of using these samples include the known existence of CNAs in the tumor samples and the availability of the paired normal samples from the same patients. We comment that BCRgt does not require paired normal samples in genotype calling, but it could achieve higher accuracy in genotyping from improved copy number assignment if paired normal samples are available (details not discussed here). The genotype calls for the paired normal samples were used to evaluate the performance of the genotype calls for the tumor samples. Note that, since tumor samples can have normal cell contamination, we excluded samples with choppy noises or poor quality, as well as those having less than 1% CNAs, in order to obtain reliable genotype calls. Thus, only 66 samples were genotyped by BCRgt.

**Figure 3:**
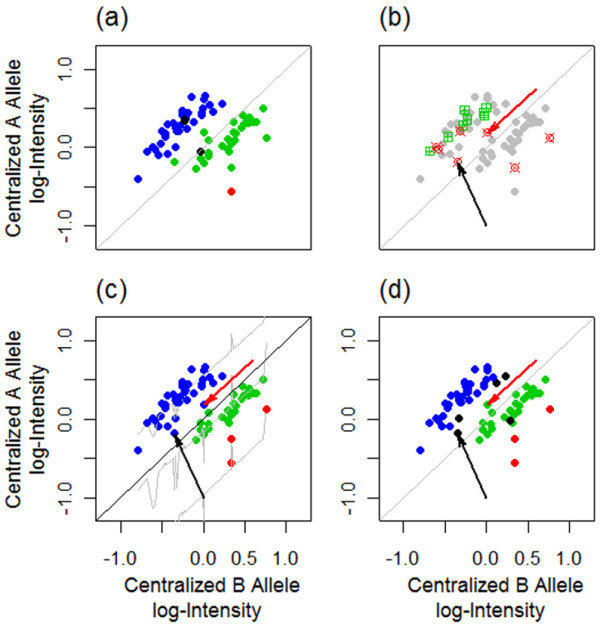
**An example of applying gtBCR on samples with CNAs. (a)** A and B allele log-intensity plot with samples colored by the genotypes of their paired normal samples: blue, green and red representing “AA”, “AB” and “BB” genotype, respectively. **(b)** The copy number status plot for each sample: red squares represent samples with copy number loss, green squares represent samples with copy number gain, and gray squares are samples without CNAs. **(c)** Genotypes called by BCRgt, with colors being the same as (a). Both the black and red arrows point to the observations that have “AB” genotype in the paired normal sample (see (a)) and have copy number loss (see (b)). They are both correctly called “AA” by BCRgt, which agrees with their copy number status. **(d)** Genotypes called by BRLMM: no call on the observation pointed by the black arrow, and “AB” call on the observation pointed by the red arrow, which is inconsistent to its copy number status.

As indicated by the three clusters in Figure 2, if there are no CNAs, samples with the same genotype, AA or AB or BB, tend to cluster together in the scattered plot of A versus B allele log-intensities. This is not the case if there are CNAs in some tumor samples. Figure 3(c) shows that it is difficult to call the observations pointed by the black and red arrows without knowing the copy number status. Note that BRLMM has no call on the observation pointed by the black arrow, and a call of AB for the observation pointed by the red arrow (Figure 3(d)). This is not consistent with the copy number status because the copy number call plot (Figure 3(b)) implies that both tumor samples have copy number loss at this SNP locus, and thus, they cannot be heterozygous. As a comparison, BCRgt is able to call both correctly as AA/A (Figure 3(c)). Since paired normal samples are available, we can use the genotypes of them as references (Figure 3(a)), to assess how copy number change affects genotype callings (see Additional file 1: Figure S3 explanation of how the intensities of two observations drop because of the copy number loss). Furthermore, we can see that among the three BB observations (colored in red, Figure 3(c)), one does not have copy number loss, while the other two do. The prediction line (the bottom gray line, Figure 3(c)) provides a good fit even though there are only three observations with the BB genotype.

**Figure 4:**
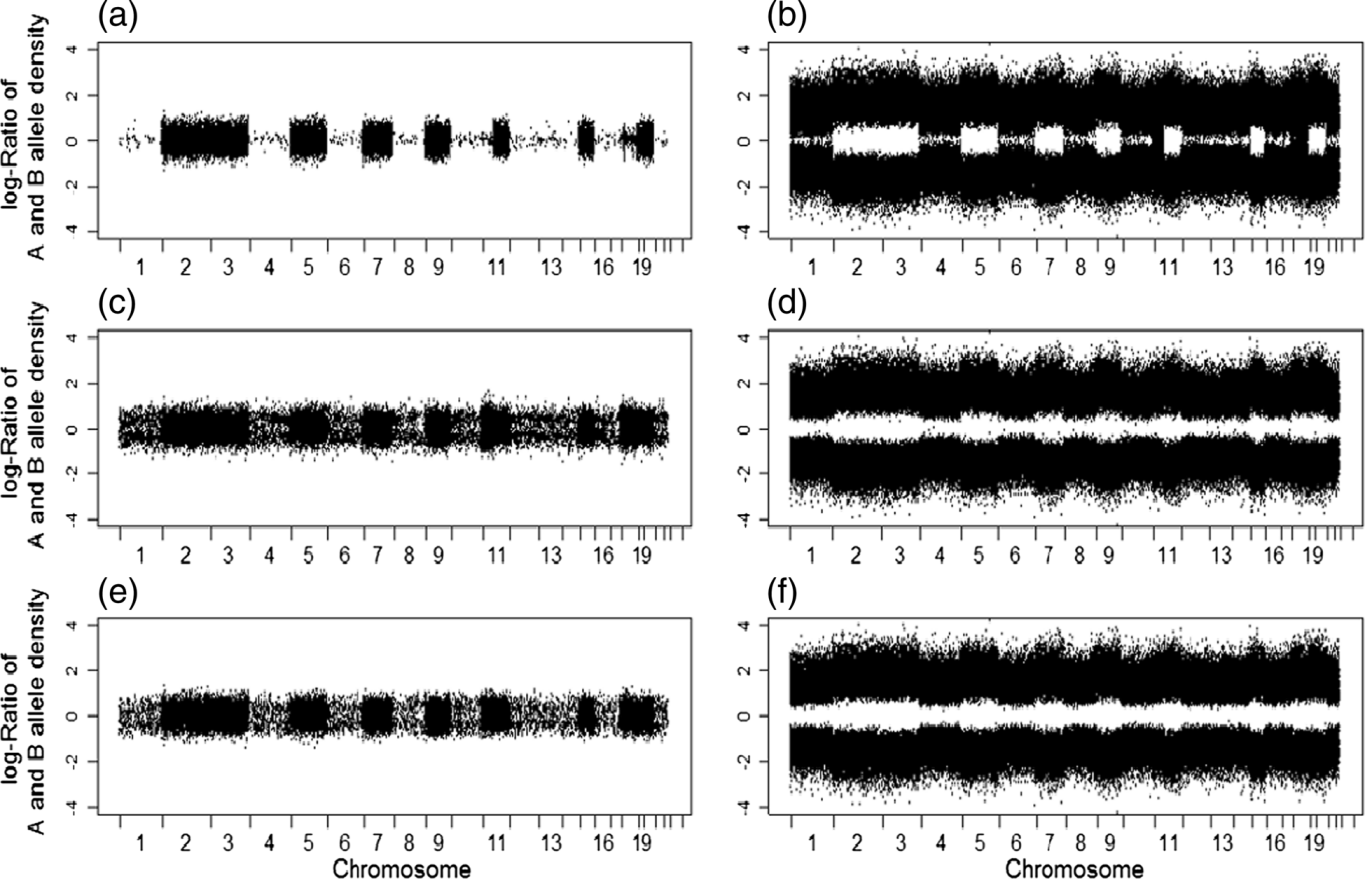
**Comparison of genotyping results generated by BCRgt, BRLMM and BCRgt with correct and incorrect copy number data.** Correct copy number data were used for **(a)-(d)**, and incorrect copy number data (we intentionally call copy number loss regions as normal regions) were used for **(e)** and **(f). (a)** SNPs with “AB” genotype called by BCRgt on a sample with multiple copy number losses on chromosomes 1, 4, 6, 8, 10, 12–14, 16–18, 21 and 22. **(b)** SNPs called “AA”/“BB” by BCRgt. **(c)** SNPs called “AB” by BRLMM. **(d)** SNPs called “AA”/“BB” by BRLMM. (e) SNPs with “AB” genotype called by BCRgt, but we intentionally misclassified the CNA status. **(f)** SNPs with “AA/BB” genotype called by BCRgt.

At the individual sample level, copy number status can be visualized on a scatter plot. We illustrate this with a sample that has multiple large segments of copy number losses (Figure 4). The copy number analysis suggested that chromosomes 1, 4, 6, 8, 10, 12–14, 16–18, 21 and 22 have copy number losses (see Additional file 1: Figure S4, or Figure 3(b) in [27]). Thus, we expect no heterozygous calls because only one copy of chromosome exists on those chromosomes. The genotype calls by BCRgt and BRLMM are presented in Figure 4. Figure 4(a) and (c) are SNPs with AB genotypes called by BCRgt and BRLMM, respectively, while Figure 4b) and (d) are SNPs with either AA or BB genotypes called by BCRgt and BRLMM separately. It is clear that on chromosomes with copy number loss, BCRgt called much less AB genotypes than BRLMM did. Note that some regions of chromosomes 11 and 18 are minor clone (the smaller population of a sample of heterogeneous tumor cells) copy number losses, but BCRgt still performs better than BRLMM on these regions. There are on average more than 4% heterozygous genotypes called by BRLMM, comparing to only 0.23% by BCRgt (Table 2) for SNPs in regions with copy number loss. We also calculated the genotyping error rates for SNPs in CNA and normal two-copy regions separately. A genotyping error occurs if the paired normal sample has AA/BB, but the tumor has AB call, or, very uncommonly, the tumor and paired normal samples have different homozygous calls. Here, we assume that the calls for the paired normal samples are highly accurate. From Table 2, we observe that in the CNA regions, the calling error rate by BRLMM is much higher (> 10 folds) than that by BCRgt, while these rates are similar in regions without CNAs. In addition, for the tumor samples with higher normal cell contamination, BRLMM generally has a much worse performance in that the proportion of AB genotypes in copy number loss regions can be as high as 13%, while the proportion by BCRgt is only around 1.5% in the same regions (see the example in Additional file 1: Figure S5).

**Table 2:** Percentages of heterozygous calls on chromosomes with copy number loss and genotyping errors (CN = copy number)

**Method**	**Heterozygous calls at CN loss region**	**Genotyping error at CN loss region**	**Genotyping error at CN normal region**
BCRgt	0.23%	0.03%	0.16%
BRLMM	4.11%	1.83%	0.28%

Misclassification of CNA status may be an issue in BCRgt because CNA status is an input covariate. However, with a retrospective check of the genotyping calls, we can recognize possible CNA global misclassification. In Figure 4(e), we intentionally misclassified the copy loss regions on chromosomes 1, 4, 6, 8, 10, 12–14, 16–18, 21 and 22 as normal, and thus the proportion of heterozygous SNPs substantially increased (in another example, Additional file 1: Figure S6, we considered the situation that the normal regions were intentionally misclassified as copy number loss regions). Based on our empirical study, we recommend that if this proportion is higher than 1%, we will examine the CNA detection algorithm. Note that this step is important not only for generating correct genotype calls, but also for examining the validity of the upstream copy number analysis. On the other hand, this type of overall misclassification is less likely if both tumor and paired normal samples are available because new statistical methods can handle this situation very well. In our recent paper on SNP array data normalization of paired samples [27], we showed that two-copy regions can be explicitly identified in the normalization process, and thus the major CNAs can be identified with very high accuracy. Note that some focal gains/losses may be missed or falsely detected. But, if true gains/losses are misidentified as normal copy number, BCRgt will perform similarly to other genotyping methods.

In regions with copy number gain, the proportion of the heterozygous calls does not provide much information because the loss of heterozygosity (LOH) does not occur in these regions. However, again because genotyping calls for the paired normal samples are available, we can calculate and compare the concordances of genotyping calls from the tumor samples with those from the paired normal samples by BCRgt and BRLMM. The results are presented in Table 3. Note that a SNP with at least one A allele and one B allele is termed as AB genotype, regardless of how many extra copies of A and/or B allele it actually has. Similarly, a homozygous SNP with at least two A alleles or two B alleles is termed as AA or BB. The overall concordance at the copy number gain regions is higher by BCRgt (99.56%) than that by BRLMM (99.14%). This reflects an approximately 1.8% increase in the concordance rate for heterozygous calls, and a slightly lower concordance (difference < 0.1%) for homozygous calls. Both calling methods give much higher (about 1 - 2%) concordance rates for homozygous calls than for heterozygous calls. Theoretically, for SNPs with AB genotype, copy number gain should not cause LOH, and should not add heterozygosity for AA or BB alleles based on the definition of LOH. Therefore, we expect a (ideally) 100% concordance rate. However, due to the issues such as normal cell contamination, the existence of minor clone, and signal saturation effect, the concordance rate is much lower compared to that of repeated arrays with the same normal samples, which often has a concordance rate of around 99.9% [7]. The (about 1 - 2%) difference between concordance rates for homozygous calls and for heterozygous calls makes perfect sense because copy number gains for homozygous calls move the intensities of the homozygous SNPs away from the heterozygous SNPs in scattered plots, as shown in Figures 1 and 2, and thus are unlikely to cause incorrect genotyping calls. On the other hand, copy number gains make heterozygous SNPs to move closer to homozygous SNPs, and consequently increase the possibility of incorrect genotyping calls.

**Table 3:** Concordance rates at copy number gain and copy number normal regions

**Method**	**Overall concordance at CN gain region**	**Heterozygous call concordance at CN gain region**	**Homozygous call concordance at CN gain region**
BCRgt	99.56%	98.76%	99.80%
BRLMM	99.14%	96.95%	99.88%

To conclude this section, we comment that including more term(s), such as the percentage of normal cell contamination, in BCRgt may help improve the concordance rate. This will be investigated in our future research.

## Conclusions

Accurate genotyping is one of the key components for a successful GWAS study. Genotyping calls for samples without CNAs can achieve a 99.5% or higher accuracy [6]. However, it remains a challenge to genotype samples with CNAs, and the result of a well-designed GWAS study can be severely compromised if the genotyping calls have low accuracy. Therefore, it is appealing to develop methods that can achieve high genotyping accuracy for samples with CNAs. Note that there are many statistical methods for analyzing copy number abnormalities. Although most of those methods work well in detecting CNAs at individual sample level, methods for formally evaluating the effect of CNAs are largely lacking.

Whether or not applying Hardy-Weinberg (HW; allele and genotype frequencies in a population will remain constant from generation to generation in the absence of other evolutionary influences) test in BCRgt should be considered case by case. For samples without CNAs, if the main assumptions of HW equilibrium are violated (for example, the violation of random mating assumption), the HW test should not be used to refine genotype calling, and a significant test result does not imply a problem in genotyping. Similarly, the HW test should not be applied when genotyping samples with CNAs because HW is not supposed to be valid for samples that are not diploid.

We achieved fairly high calling accuracy for samples without CNAs when directly applying BCRgt to all 270 HapMap samples. Later, a training step was added to further improve the accuracy. We adopted a strategy similar to the cross-validation approach – half of the 270 samples were randomly selected and used to train the model, while the remaining half were used as the validation set to calculate the concordance rates with HapMap call. We comment that this strategy is unlikely to overestimate the concordance rate because we avoided using the same biological sample in both the training and validation sets. Note that there are other strategies that may work better. For example, we can train the data produced by one laboratory for a subset of the HapMap samples, and then apply the trained model to the data obtained from a different laboratory for the complementary subset of HapMap samples. By so doing, both biological samples and experimental variations are independent between the training and validation sets.

BCRgt has substantial improvement over BRLMM for calling SNPs in copy number loss regions, and performs better in copy number gain regions. There is minimal difference between these two methods in calling SNPs in normal two copy regions.

The core of BCRgt is a Bayesian linear regression, which allows the adjustment for CNAs in SNP calling. It is also feasible to incorporate additional information, such as the percentage of normal cell contamination, into the model for better genotype calling results. Moreover, incorporating genotypes of the paired normal samples into the model might further reduce genotyping error should those data be available.

Depending on objectives, the output format of genotype calls for SNPs in the CNA regions can be flexible. For example, genotypes in the copy number loss regions can be outputted as 0/A/B, or 00/AA/BB, and similarly those in one copy gain regions can be outputted as AAA/AAB/ABB/BBB, or AA/AB/BB. We do not suggest assigning higher copy number calls to the genotypes in copy number gain regions due to several issues including: 1) normal cell contamination, 2) array signal saturation effect, 3) less robustness of more complex models, and 4) increased difficulty in result interpretation.

Unlike the Affymetrix platform, Illumina arrays produce two measurement variables, log R Ratio and B Allele Frequency (BAF; a normalized measure of relative signal intensity of the B and A alleles). Statistical methods, based on either the Bayesian approach or HMM, have been proposed to utilize both variables simultaneously to perform the analysis. However, most of these methods, such as PennCNV [28], QuantiSNP [29] and OncoSNP [30], were designed mainly for detecting CNA/CNV, and are not applicable to make genotyping calls by default. GenoSNP [11], for an Illumina platform too, provided a very low homozygosity rate (about 90%) when used for genotyping deletion regions in the HapMap samples [28,31]. In addition, though PennCNV has a plug-in for calculating BAF for Affymetrix data, the QN component used in the process makes it unattractive for samples with CNAs (it has been shown that QN is not optimal for handling samples with CNAs).

Most of the current genotyping methods assume bivariate normality on the joint distribution of A and B alleles for each genotype, which implicitly assumes that the marginal distribution of each allele is unimodal. This may not be true if CNAs exist because the marginal distribution may be bimodal. In contrast, BCRgt adopts a linear regression approach. Thus the assumption we need is that the residuals of each cluster follow a normal distribution, which is commonly made in regression analysis.

Alhough accurate copy number calls are required by BCRgt, at each SNP, an incorrect copy number call for one sample does not affect genotyping calls for other samples even though this property will not hold if the majority of the copy number calls are incorrect. In sum, BCRgt requests that the extra information added to the regression model is of high quality, but this should not be a big concern if paired normal samples are available. If the paired normal samples are not available, how to obtain high quality information from quality array and cytogenetic data is worth a lot of discussion. This is out of the scope of this paper.

### Availability

The R package “BCRgt” including documentation is available online. See the website, http://publichealth.lsuhsc.edu/BCRgt.html for details.

## Competing interests

The authors declare that they have no competing interests.

## Authors’ contributions

SY and ZF designed the experiments. SY did the data analysis. SY, ZF and XC wrote the paper. All authors read and approved the final manuscript.

## Supplementary Material

Additional file 1: Figure S1 A brief derivation of the posterior distribution for β_s._**S2**. Cluster regression parameter estimation. **Figure S1.** An example of “BB” genotype missing (SNP #12). **Figure S2.** Illustration of the difference in absolute change in signal intensity between copy number gain and loss. **Figure S3.** Illustration of copy number loss caused signal intensity drop. **Figure S4.** Copy number status of the same sample presented in Figure 4. **Figure S5.** (1) An example of higher normal cell contamination. **Figure S5.** (2) The copy number status of the sample presented in **Figure S5. Figure S6.** An example that the normal regions were intentionally misclassified as copy number loss regions. Click here for file
