# Supplementary material for: BCRgt: a Bayesian cluster regression-based genotyping algorithm for the samples with copy number alterations
Source: BMC Bioinformatics. 2014 Mar 15;15:74. doi: 10.1186/1471-2105-15-74 (PMC4003822; doi:10.1186/1471-2105-15-74)
Supplement: Additional file 1: Figure S1 — A brief derivation of the posterior distribution for βs.S2. Cluster regression parameter estimation. Figure S1. An example of “BB” genotype missing (SNP #12). Figure S2. Illustration of the difference in absolute change in signal intensity between copy number gain and loss. Figure S3. Illustration of copy number loss caused signal intensity drop. Figure S4. Copy number status of the same sample presented in Figure 4. Figure S5. (1) An example of higher normal cell contamination. Figure S5. (2) The copy number status of the sample presented in Figure S5. Figure S6. An example that the normal regions were intentionally misclassified as copy number loss regions. [file 1471-2105-15-74-S1.doc]

**Supplementary file**

**S1. A brief derivation of the posterior distribution for β_s._**

The posterior mean of β.

Normal-inverse-gamma distribution was used as the prior distribution, with density $p\left(\mathbf{\beta}, \sigma^{2}\right)=p\left(\mathbf{\beta} | \sigma^{2}\right)p\left( \sigma^{2}\right)$. Further, instead of using a prior on *σ^2^*, we will use the frequentist method to estimate *σ^2^*. Consequently, the prior is reduced to a Multi-normal distribution as $N\left(\mathbf{\beta}_{prior},\text{ }\sigma^{2}\mathbf{V}\right)$, with the elements, $\beta_{k0-prior}$ and , of the mean vector representing the expected values of the distributions of the intercept and slope for cluster *k*, respectively.

With some routine algebraic steps, we have the posterior distribution as follows.

$$\begin{matrix} p\left(\mathbf{\beta}|y,\sigma^{2}\right)\propto p\left(\mathbf{\beta}|\sigma^{2}\right)p\left(\text{ }y|\mathbf{\beta},\text{ }\sigma^{2}\right) \\ \text{ }=e^{-\frac{\left(\mathbf{\beta}-\mathbf{\beta}_{prior}\right)'\mathbf{V}^{-1}\left(\mathbf{\beta}-\mathbf{\beta}_{prior}\right)}{2\sigma^{2}}}e^{-\frac{\left(y-\mathbf{X\beta}\right)'\left(y-\mathbf{X\beta}\right)}{2\sigma^{2}}} \\ \text{ }=e^{-\frac{y'y-y'\mathbf{X\beta}-\left(\mathbf{X\beta}\right)'y+\mathbf{\beta'X'X\beta}+\mathbf{\beta'V}^{-1}\mathbf{\beta}-\mathbf{\beta'V}^{-1}\mathbf{\beta}_{prior}-\mathbf{\beta}_{prior}'\mathbf{V}^{-1}\mathbf{\beta}-\mathbf{\beta}_{prior}'\mathbf{V}^{-1}\mathbf{\beta}_{prior}}{2\sigma^{2}}} \\ \text{ = }c_{1}e^{-\frac{\mathbf{\beta}'\left(\mathbf{X'X}+\mathbf{V}^{-1}\right)\mathbf{\beta}-\left(y'\mathbf{X}+\mathbf{\beta}_{prior}'\mathbf{V}^{-1}\right)\mathbf{\beta}-\mathbf{\beta}'\left(\mathbf{X}'y+\mathbf{V}^{-1}\mathbf{\beta}_{prior}\right)}{2\sigma^{2}}} \\ \text{ = }c_{2}e^{-\frac{\left(\mathbf{\beta}-\left(\mathbf{V}^{-1}+\mathbf{X'X}\right)^{-1}\left(\mathbf{X}'y+\mathbf{V}^{-1}\mathbf{\beta}_{prior}\right)\right)^{'}\left(\mathbf{V}^{-1}+\mathbf{X'X}\right)\left(\mathbf{\beta}-\left(\mathbf{V}^{-1}+\mathbf{X'X}\right)^{-1}\left(\mathbf{X}'y+\mathbf{V}^{-1}\mathbf{\beta}_{prior}\right)\right)}{2\sigma^{2}}}, \end{matrix}$$

where $c_{1},\text{ }c_{2}$ are two quantities, free of $\mathbf{\beta}$. Thus,

$$\begin{matrix} p\left(\mathbf{\beta}|y,\sigma^{2}\right)\text{ \textasciitilde}N\left(\left(\mathbf{V}^{-1}+\mathbf{X'X}\right)^{-1}\left(\mathbf{V}^{-1}\mathbf{\beta}_{prior}+\mathbf{X}'y\right),\left(\mathbf{X'X}+\mathbf{V}^{-1}\right)^{-1}\sigma^{2}\right),\text{ and} \\ \hat{\mathbf{\beta}}_{Bayes}=\left(\mathbf{V}^{-1}+\mathbf{X'X}\right)^{-1}\left(\mathbf{V}^{-1}\mathbf{\beta}_{prior}+\mathbf{X}'y\right) \end{matrix}$$

**S2. Cluster regression parameter estimation**

We assume that the (*n×2*) matrix ***X*** *= (****1****_n_,* ***x****)* is fixed, where ***1****_n_* is the all-ones vector, ***x*** is the vector of the observed A allele log-intensities, and *n* is the total number of samples. Let $Y=(Y_{1},Y_{2},\ldots,Y_{n})^{T}$be the vector of the observed B allele log-intensities, correspondingly. Denote **β**, σ^2^ and π as unknown parameters. In addition, $C=\left\{C_{1},C_{2},\ldots,C_{n}\right\}$are the latent data, where $C_{i}\in\{1, 2, ..., K\}$ is the cluster indicator for the *i^th^* sample, and *K i*s the number of clusters. Denote $\pi_{k}$ as the probability that a sample belongs to the cluster *k* and $\pi=(\pi_{1},\cdots,\pi_{K}).$ Then $C_{i}\sim Multinomial\left(\pi\right)\text{, }$and the joint density of *Y* and *C* is,

$$\begin{matrix} f\left(Y,C|\mathbf{X};\mathbf{\beta},\sigma^{2},\pi\right) \\ =f\left(C|\mathbf{X};\mathbf{\beta},\sigma^{2},\pi\right)f\left(Y|\mathbf{X},C;\mathbf{\beta},\sigma^{2},\pi\right) \\ =f\left(C|\pi\right)f\left(Y|\mathbf{X},C;\mathbf{\beta},\sigma^{2}\right), \end{matrix}$$

where the last equality holds due to the facts that *C* depends on π only and that *Y* is independent of *π*, given *C*. Thus the full log likelihood is,

$$\begin{matrix} l(\mathbf{\beta},\sigma^{2},\pi;y,C|\mathbf{X})=\sum_{i=1}^{n} \sum_{k=1}^{K} \left[l(\pi;C)+l(\mathbf{\beta},\sigma^{2};y|\mathbf{X})\right] \\ \text{ }=\sum_{i=1}^{n} \sum_{k=1}^{K} C_{ik}\log(\pi_{k})-\sum_{i=1}^{n} \sum_{k=1}^{K} C_{ik}\left[\frac{1}{2}\log(\sigma_{k}^{2})+\frac{\left(y_{i}-x_{i}^{T}\beta_{k}\right)^{2}}{2\sigma_{k}^{2}}\right]\text{ ,} \end{matrix}$$

where $C_{ik}=1,\text{ if }C_{i}=k,\text{ or }0\text{ otherwise.}$

***The Estimation step:***

After setting the initial values of **β**, σ^2^ and π, we iteratively find the expectation of the log likelihood function conditional on all data and the estimated parameters from the previous iteration: . In fact, to maximize this conditional expectation, it is sufficient to maximize $E\left(C_{ik}=1|\mathbf{\beta},\text{ }\sigma^{2},\text{ }\pi,y\right)$because is linear in $C_{ik}.$

By Bayesian theory, after the iteration *t*, $C_{ik}$ is estimated by,

where $\pi_{k}^{(t)}$is the probability of an observation in Cluster *k* at iteration *t*, and is normal density function, conditional on

***The Maximization step:***

With $\sum_{k=1}^{K} \pi_{k}=1$, we need to update the parameters to maximize :

$$\begin{matrix} \hat{\pi}_{k}^{(t+1)}=\sum_{i=1}^{n} \hat{C}_{ik} \\ \hat{\beta}_{k}^{(t+1)}=\left(X'\hat{c}_{k}\hat{c}_{k}^{T}X\right)^{-1}\left(X'\hat{c}_{k}\hat{c}_{k}^{T}y\right), \end{matrix}$$

where $\hat{c}_{k}=(\hat{C}_{1k},\hat{C}_{2k},...,\hat{C}_{nk})^{T}$. By adding the prior, we have

$$\hat{\beta}_{k}^{(t+1)}=\left(V_{k}^{-1}+X'\hat{c}_{k}\hat{c}_{k}^{T}X\right)^{-1}\left(V_{k}^{-1}\beta_{prior-k}+X'\hat{c}_{k}\hat{c}_{k}^{T}y\right).$$

Note that, for the cluster without any observation, the estimation is the same as the prior. In order to use all the samples for a stable variance estimate, we used a pooled estimation,

where z = 1 if *k* = 2, or 2 otherwise.

Repeat the above Expectation and Maximization steps until a converge criteria is met (we used ), or the iteration number reaches 30.

**Figure S1. An example of "BB" genotype missing (SNP #12).**


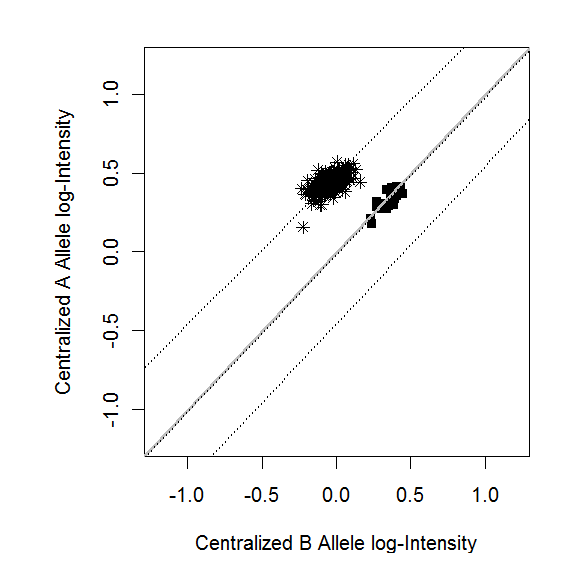


There is no BB genotype sample for this SNP, however, BCRgt correctly identified the AA and AB genotype samples because of the Bayesian prior provided sufficient information in assigning the correct genotype calls to each cluster.

**Figure S2. Illustration of the difference in absolute change in signal intensity between copy number gain and loss.**


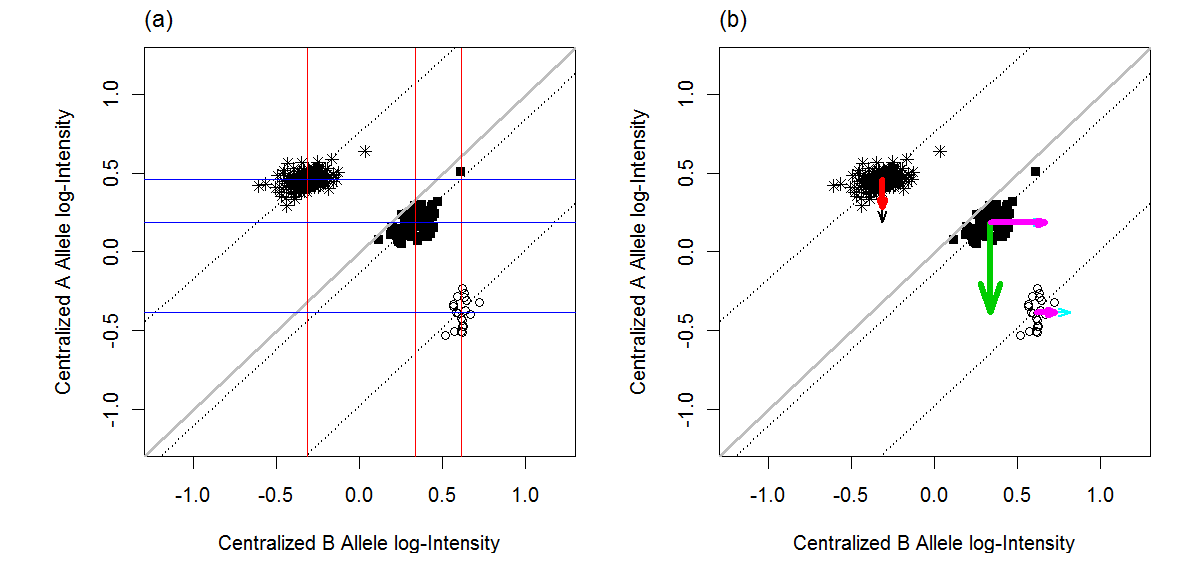


(a) The vertical lines (red) from left to right represent 0, 1 and 2 copies of B allele, respectively. Note that the increase in log-intensity from one copy of B allele to 2 copy is much less than from no B allele to one copy of B allele. (b) Because of array signal saturation effect, once a homozygous SNP losses one copy of an allele, the magnitude of decrease in signal intensity is less than the theoretical prediction, for example, the bold red, comparing the narrower black representing the theoretical value in decrease. On the other hands, when a SNP has allele gains, the magnitude of increase of signal intensity is less than the theoretical prediction, for example, the Cyan arrows are the theoretical prediction of one copy B allele gain for "AB" and "BB" genotypes, and in practice, the magnitude of increase is less (the bold pink arrows). In addition, the saturation effect would be less significant for losses of heterozygous SNPs when normal sample contamination is not considered (see the bold green line).

**Figure S3. Illustration of copy number loss caused signal intensity drop.**


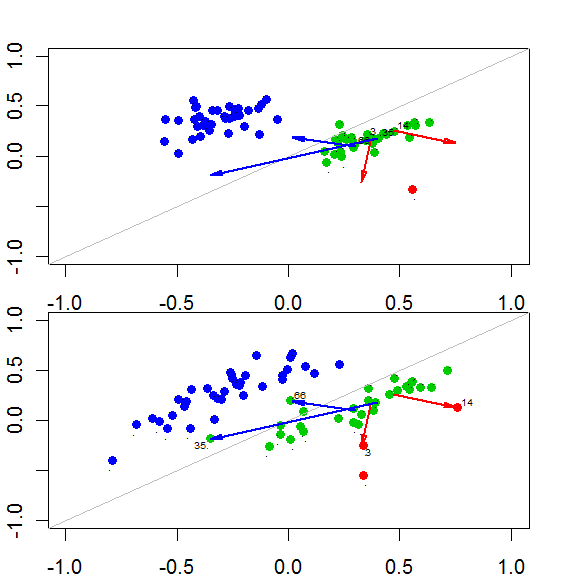


A real example of how copy number loss causes signal intensity drops. Four samples, #3, #14, #35 and #66, have one copy of allele loss. (a) The starting points of the arrows are the A and B allele intensities of the paired normal samples. (b) The corresponding tumor samples. Samples #3 and #14 lost one-copy of A allele, and samples #35 and #66 lost one-copy of B allele.

**Figure S4. Copy number status of the same sample presented in Figure 4.**


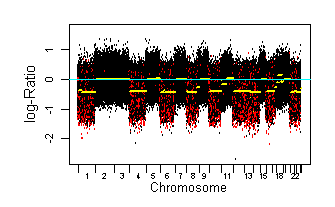


Probes that have AB genotype calls in normal, but AA or BB in tumor, were highlighted in red; probes that do not have this change are still in black. Since most of the probes with heterozygous loss are located in chromosomes 1, 4, 6, 8, 10, 12-14, 16, 17, 21, 22, we assume that those are the one-copy loss chromosomes.

**Figure S5. (1) An example of higher normal cell contamination.**


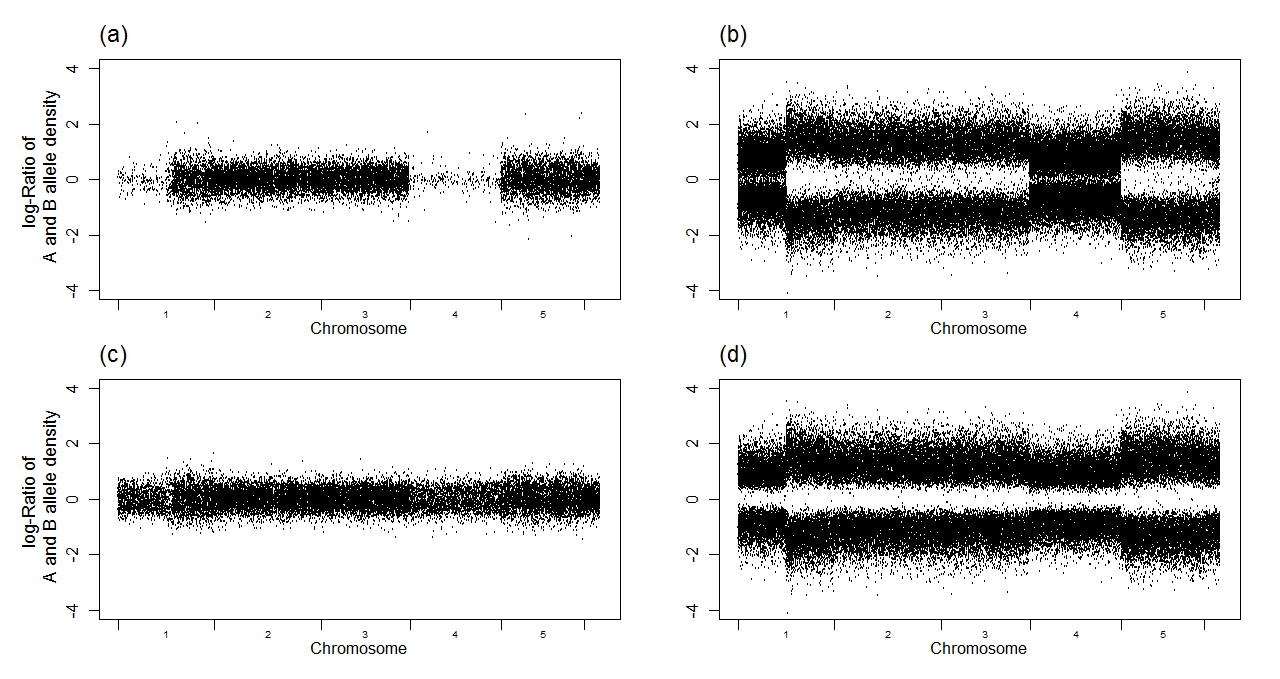


(a) SNPs with "AB" genotype called by BCRgt on a sample (only chromosomes 1-5 are presented for better resolution). (b) SNPs called "AA"/"BB" by BCRgt. (c) SNPs called "AB" by BRLMM. (d) SNPs called "AA"/"BB" by BRLMM.

**Figure S5. (2) The copy number status of the sample presented in Figure S5.**


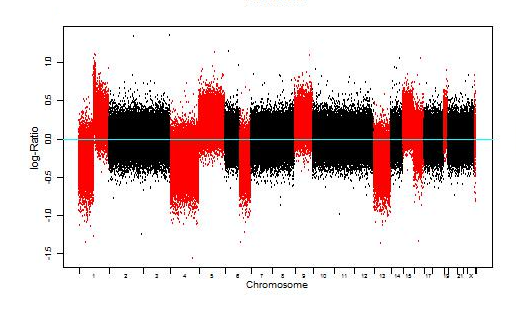


The observations in red are the SNPs that have copy number alterations, and those in black are the normal two-copy SNPs.

**Figure S6. An example that the normal regions were intentionally misclassified as copy number loss regions.**


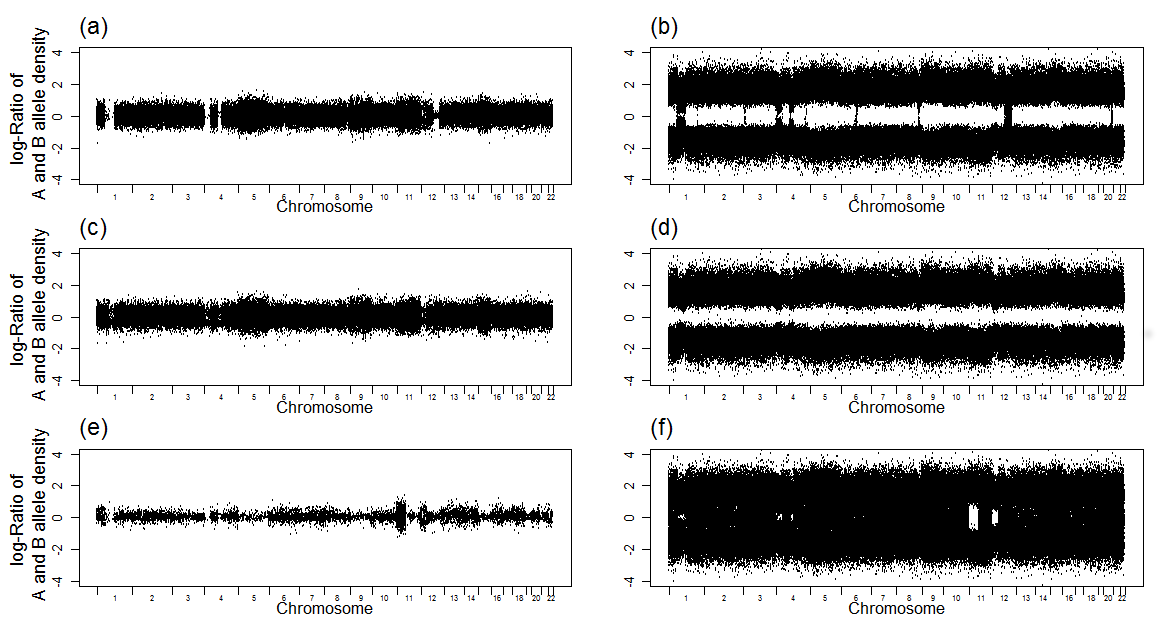


Compare genotyping results generated by BCRgt, BRLMM and BCRgt with correct and incorrect copy number data. Correct copy number data were used for (a)-(d), and incorrect copy number data (we intentionally call copy number normal regions as copy number loss regions) were used for (e) and (f). (a) SNPs with "AB" genotype called by BCRgt. (b) SNPs called "AA"/"BB" by BCRgt. (c) SNPs called "AB" by BRLMM. (d) SNPs called "AA"/"BB" by BRLMM. (e) SNPs with "AB" genotype called by BCRgt, but we intentionally misclassified the CNA status. (f) SNPs with "AA/BB" genotype called by BCRgt.
